# Novel methodologies for host-microbe interactions and microbiome-targeted therapeutics in 3D organotypic skin models

**DOI:** 10.1186/s40168-023-01668-x

**Published:** 2023-10-17

**Authors:** Gijs Rikken, Luca D. Meesters, Patrick A. M. Jansen, Diana Rodijk-Olthuis, Ivonne M. J. J. van Vlijmen-Willems, Hanna Niehues, Jos P. H. Smits, Peter Oláh, Bernhard Homey, Joost Schalkwijk, Patrick L. J. M. Zeeuwen, Ellen H. van den Bogaard

**Affiliations:** 1 Department of Dermatology, Radboud University Medical Center (Radboudumc), Nijmegen, The Netherlands; 2 Department of Dermatology, University Hospital Düsseldorf, Medical Faculty, Heinrich-Heine-University Düsseldorf, Düsseldorf, Germany

**Keywords:** Keratinocytes, Organoids, Colonization, Microbiota, Antibiotics

## Abstract

**Background:**

Following descriptive studies on skin microbiota in health and disease, mechanistic studies on the interplay between skin and microbes are on the rise, for which experimental models are in great demand. Here, we present a novel methodology for microbial colonization of organotypic skin and analysis thereof.

**Results:**

An inoculation device ensured a standardized application area on the *stratum corneum* and a homogenous distribution of bacteria, while preventing infection of the basolateral culture medium even during prolonged culture periods for up to 2 weeks at a specific culture temperature and humidity. Hereby, host-microbe interactions and antibiotic interventions could be studied, revealing diverse host responses to various skin-related bacteria and pathogens.

**Conclusions:**

Our methodology is easily transferable to a wide variety of organotypic skin or mucosal models and different microbes at every cell culture facility at low costs. We envision that this study will kick-start skin microbiome studies using human organotypic skin cultures, providing a powerful alternative to experimental animal models in pre-clinical research.

Video Abstract

**Supplementary Information:**

The online version contains supplementary material available at 10.1186/s40168-023-01668-x.

## Introduction

The skin is a multi-faceted barrier organ that hosts a diversity of commensal microbial communities, composing the human skin microbiota. Over the past decade, we have witnessed a scientific breakthrough with respect to our knowledge and understanding of these microorganisms due to advances in sequencing technologies and the initiation of the human microbiome project [1]. Skin microbiome composition and diversity varies between body sites and individuals and is affected by environmental influences [2, 3]. The most abundant bacteria identified at the genus level are *Corynebacterium*, *Cutibacterium*, and *Staphylococcus* [2, 4], along with the most common fungal commensal *Malassezia* [4–6]. These microbes play an important role in skin health by educating the immune system [7–9], preventing the colonization by pathogens [10, 11], and promoting skin barrier function [12, 13].

Alterations in skin microbiome composition, called dysbiosis, are nowadays associated with a plethora of skin conditions, such as atopic dermatitis (AD), psoriasis, and acne [14–21]. Colonization and infection of the skin by *Staphylococcus aureus* (*S. aureus*) has been under investigation for decades [22, 23], but recent studies also suggest other *Staphylococcus* species like *S. epidermidis* [24] and *S. capitis* [25] to contribute to skin pathologies. The question remains whether dysbiosis is the cause or consequence of skin diseases and to what extent the microbiome can be leveraged as a therapeutic target [26–28]. Following initial descriptive studies on the skin microbiome [4, 29], investigative mechanistic studies using biologically relevant experimental models are of utmost importance to dissect the cause or contribution of microbial dysbiosis to health and disease [27, 30, 31].

Notwithstanding the importance and utility of widely used in vivo-animal models [32–34], the skin microbiome of rodents is significantly different from humans and the instability of the microbiome in laboratory animals is known to affect the experimental outcome [30]. Alternatively, human skin cell cultures (e.g., keratinocyte monolayer cultures) allow investigations on the direct interaction between keratinocytes and microbes [35, 36]. Herein, cultures inoculated with live bacteria are restricted to be short-term as cell viability will be compromised upon the bacterial overgrowth within a few hours [37, 38]. Optionally, heat-killed bacteria, bacterial components, or the bacterial culture supernatant can be used [39–41]. However, these do not mimic the actual colonization onto the protective *stratum corneum*, which acts as a physical barrier and filter for microbial metabolites [42]. Investigative studies on these metabolites and potential quorum sensing molecules [43, 44] that interact with bacterial or host cell receptors to activate signal transduction pathways [13, 45, 46], would benefit from models in which live bacteria are grown under biologically relevant culture conditions, such as a natural growth substrate (the *stratum corneum*) with a viable epidermis underneath.

Advanced organotypic skin models (either full-thickness skin or epidermal equivalents) have recently been used more often in host-microbe interaction studies. Next to bacterial infection models, microbial colonization is reported for a variety of skin-related bacteria and fungi. To summarize the current state-of-the-art, we provide a literature overview including experimental details and read-out parameters in Supplemental Table S1. These studies clearly indicate the utility of organotypic skin models for skin microbiome research, but also highlight a lack of standardization, relatively short culture periods of up to 24 h, the high risk of basolateral culture infections and low assay throughput at high costs. Furthermore, the common use of standard cell culture conditions (37 °C at a high relative humidity) in these microbial exposed culture studies favors the growth of aerobic bacteria which will affect the bacterial diversity of *in vitro* cultured skin microbiome samples [47].

In an attempt to overcome these limitations, we here present a low cost and easy to use technical advance for microbial colonization of 3D human epidermal equivalents (HEEs). This may enable standardization of microbiome research using organotypic skin models and facilitate multi-parameter analytics from one single culture. Using this model system we provide proof-of-concept for differential host defense responses by skin commensals and pathogens, establish long-term culture periods up to 2 weeks and implement effective intervention studies by topical antibiotics.

## Extended methods description

Resources table

**Table:** 

Reagent or resource	Source	Identifier
Antibodies (Supplemental Table S2)		
Mouse monoclonal anti-Filaggrin (clone FLG01)	Thermo Fisher Scientific	Cat#FLG01-1 RRID:AB 2894828
Rabbit monoclonal anti-Ki67 (clone SP6)	Abcam	Cat#ab16667, RRID:AB 302459
Mouse monoclonal anti-Involucrin (clone Mon150)	[48]	N/A
Mouse monoclonal anti-Keratin 10 (clone DE-K10)	Abcam	Cat# ab9026 RRID:AB 306950
Rabbit monoclonal anti-SKALP/Elafin (clone 92-1)	[49]	N/A
Goat polyclonal anti-hBD2	Abcam	Cat# ab9871, RRID:AB 296681
Bacterial and virus strains (Supplemental Table S3)		
* Cutibacterium acnes*	ATCC	ATCC-6919
* Staphylococcus epidermidis*	ATCC	ATCC-12228
* Staphylococcus capitis*	Clinical isolate	N/A
* Corynebacterium aurimucosum*	Clinical isolate	N/A
* Staphylococcus aureus*	ATCC	ATCC-29213
* Staphylococcus aureus*	Clinical isolate from AD skin (SA-DUS-011)	N/A
Biological samples		
N/A		
Chemicals		
CnT-Prime Epithelial Proliferation Medium	CELLnTEC	Cat#CnT-PR
CnT-Prime 3D Barrier Culture Medium	CELLnTEC	Cat#CnT-PR-3D
EpiLife Medium, with 60 µM calcium	Gibco	Cat#MEPI500CA
Dulbecco′s Modified Eagle′s Medium - high glucose	Sigma-Aldrich	Cat#D6546
Formaldehyde solution 4%, buffered, pH 6.9	Sigma-Aldrich	Cat#1.00496
Fusidic acid sodium salt	Sigma-Aldrich	Cat#F0881
Fluoromount-G™ Mounting Medium, with DAPI (4′,6-diamidino-2-phenylindole)	Thermo Fisher Scientific	Cat#00-4959-52
Critical commercial assays		
Vectastain ABC Kit (Rabbit, Mouse, Goat IgG)	Vectorlabs	Cat#PK-6101, 6102, 6105
Deposited data		
N/A		
Experimental models: cell lines		
Human: Primary normal keratinocytes	[50]	N/A
Human: N/TERT-2G	[51, 52]	N/A
Experimental models: organisms/strains		
N/A		
Oligonucleotides (Supplemental Table S4)		
Primers for human beta defensin-2 (hBD2), *DEFB4*	This paper	N/A
Primers for ribosomal phospoprotein P0, *RPLP0*	This paper	N/A
Primers for chemokine (C-C motif) ligand 20, *CCL20*	This paper	N/A
Primers for interleukin-1β, *IL1B*	This paper	N/A
Primers for S100 calcium-binding protein A9, *S100A9* (also known as migration inhibitory factor-related protein 14, *MRP14*)	This paper	N/A
Recombinant DNA		
N/A		
Software and algorithms		
GraphPad Prism 9.0		
Other		
Glass Culture Cylinders, 4 mm inner diameter, 5 mm height	Bioptechs	Cat#070303-04
Nunc Cell Culture Inserts in 24-well Carrier Plate Systems, 0.4 micron pore size	Thermo Fisher Scientific	Cat#141002

## Contact for reagent and resource sharing

Further information and requests for resources and reagents should be directed to and will be fulfilled by the lead contact, Ellen van den Bogaard (Ellen.vandenBogaard@radboudumc.nl).

## Experimental model and method details

### Primary keratinocyte isolation

Surplus human skin was obtained from plastic surgery (according to the principles of the Declaration of Helsinki). Human primary keratinocytes were isolated as previously described [50]. Briefly, 6-mm full-thickness biopsy punches of the freshly excised skin tissue were taken and placed into antibiotic/antimycotic medium for 4 h at 4 °C. Thereafter, 0.25% trypsin in phosphate buffered saline (PBS) was added and incubated overnight (o/n) at 4 °C. Next, the enzymatic reaction was stopped by the addition of 10% (v/v) fetal bovine serum (GE Healthcare Life Sciences). A pair of tweezers was used to scrape the surface of the biopsy for harvesting of the keratinocytes. The keratinocytes were counted and seeded onto feeder cells at a density of 50,000 cells/cm^2^ in keratinocyte growth medium. The cells were harvested at 95% confluency with a final DMSO concentration of 10% and the cryovials were placed o/n into a freezing container at − 70 °C, after which the cells were stored in liquid nitrogen.

### 3D human epidermal equivalent (HEE) culture

**Fig. 1:**
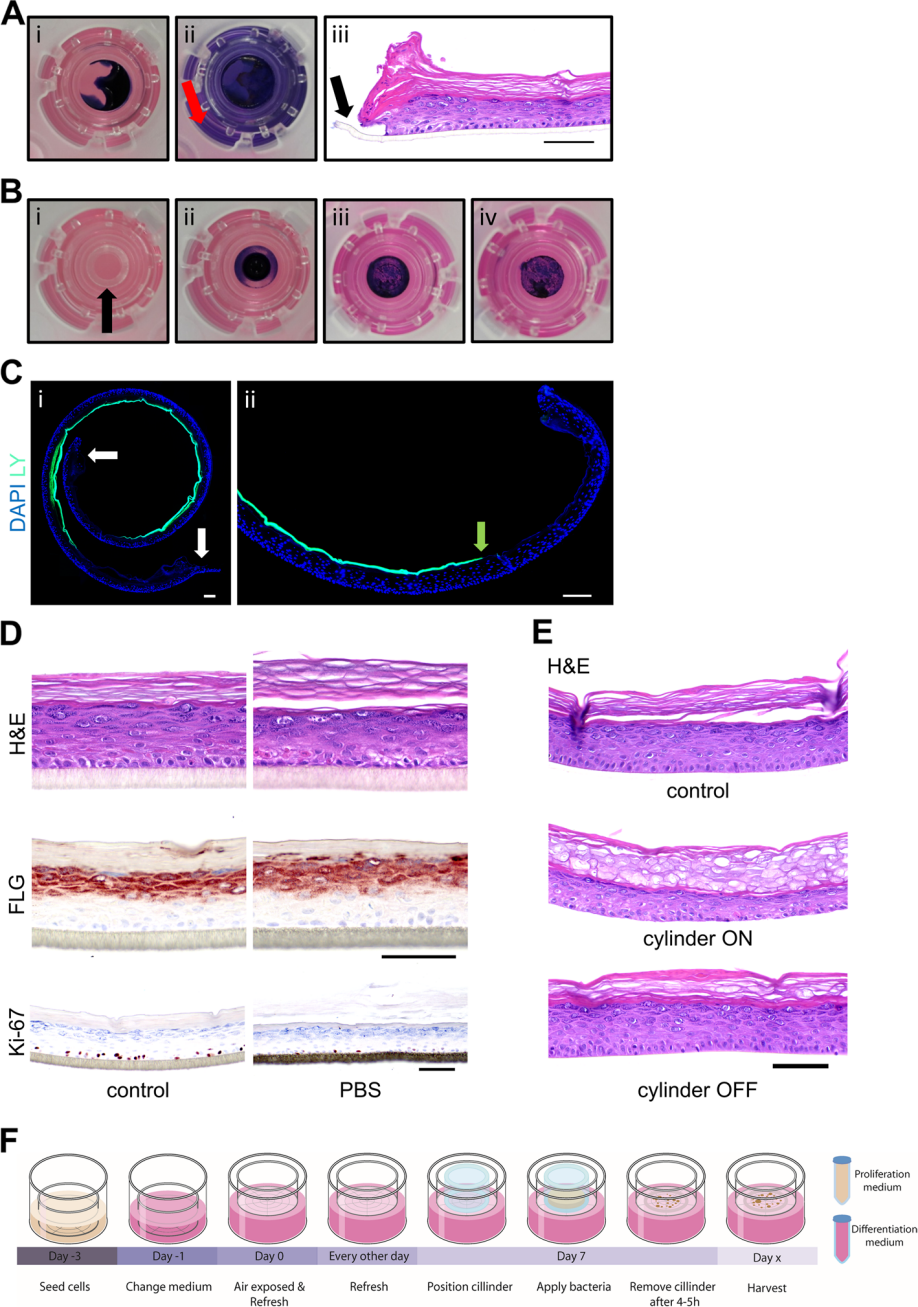
Validation of glass cylinder methodology.** A** (i) 25 µL drop of trypan blue in PBS applied on top of the HEE, (ii) the basolateral penetration of trypan blue after 4 h of incubation (red arrow), and (iii) H&E staining showing the open edges of the HEE (black arrow). **B** (i) Glass cloning cylinder on top of the HEE indicated with the black arrow, (ii) 25 µL of trypan blue in PBS was pipetted inside the cylinder, (iii) the PBS was evaporated 4 h later (in flow cabinet on heated plate at 37 °C, without lid), and (iv) the removal of the cylinder revealed a blue colonized circle without basolateral penetration. **C** Lucifer yellow (LY) added inside the glass cylinder and harvested after 2.5 h of incubation. DAPI staining and fluorescent imaging (× 10 magnification) shows (i) the distribution of LY onto the whole HEE and (ii) clean edges. **D** H&E, Ki-67, and filaggrin (FLG) staining of HEE with a drop of PBS on top for 24 h to analyze the morphological changes and protein expression patterns compared to the control. **E** Difference in morphology between the removal of the cylinder after PBS evaporation or leaving it on top of the HEE for 48 h shown with an H&E staining. **F** Schematic overview of HEE culture and the topical application of bacteria using a glass cylinder. Scale bar = 100 µm

HEEs were generated according to the protocols previously described (Rikken et al. 2020). Briefly, cell culture inserts (24-wells, 0.4 µm pore size filters; Thermo Fisher Scientific, Nunc) were coated with 150 µL of rat tail collagen in sterile cold PBS (100 µg/mL, BD Biosciences, Bedford, USA) at 4 °C for 1 h. Thereafter, excessive collagen solutions were carefully aspirated and the filters were washed with sterile cold PBS. Then, 150,000 primary human keratinocytes were seeded submerged in 150 µL CnT-prime medium (CELLnTEC, Bern, Switzerland). Nine hundred microliters of CnT-prime was added to the basolateral chamber, after which the cultures were incubated at 37 °C and 5% CO_2_. After 48 h, cultures were switched to 3D differentiation medium, which consists of 60% CnT-Prime 3D Barrier medium (CELLnTEC, Bern, Switzerland) and 40% high glucose Dulbecco’s modified Eagle’s medium (DMEM, D6546, Sigma-Aldrich). Twenty-four hours later, the HEEs were lifted to the air-liquid interface (ALI) using 1600 µL of 3D differentiation medium, which was refreshed every other day. The HEE culture schedule is depicted in Fig. 1F (created with Adobe Illustrator, https://www.adobe.com/illustrator).

For the N/TERT-2G cells, EpiLife medium (Gibco) or CnT-prime (CELLnTEC) was used (based on availability) for seeding the cells and during the first 48 h of submerged culture. The N/TERT-2G HEEs were generated from N/TERT-2G keratinocytes at passage 3.

### Bacterial cultures

Bacterial strains (see Supplemental Table S2) were obtained from the Department of Medical Microbiology of the Radboud University Medical Center and the Department of Dermatology of the Heinrich-Heine-University in Düsseldorf (clinical isolate of AD skin, SA-DUS-011). *S. aureus*, *S. epidermidis*, *S. capitis*, and *Corynebacterium aurimucosum* (*C. aurimucosum*) strains were grown o/n on Columbia agar with 5% sheep blood (Becton, Dickinson and Co.) under aerobic conditions at 37 °C. Single colonies were used to inoculate cultures in 3 mL brain heart infusion (BHI) medium (Mediaproducts BV) in a 14-mL round bottom tube with snap cap (Cat#352057, Falcon, Corning) and incubated o/n at 37 °C while shaking (225 rpm). Thereafter, bacterial cultures were diluted 100 times (30 µL in 3 mL BHI medium) and grown for another 2.5 h in a shaking incubator to reach exponential growth. *Cutibacterium acnes* (*C. acnes*) was grown on Columbia agar with 5% sheep blood for 2 days at 37 °C under anaerobic conditions (anaerobic jar system with an Oxoid Anaerogen 3.5 L sachet (Cat#AN0035A, Thermo Fisher Scientific)), after which a single colony was picked and cultured o/n in 3 mL BHI medium at 37 °C under anaerobic conditions. Thereafter, the bacteria were collected by centrifugation. The pellets containing the bacteria were washed twice in PBS and finally resuspended in PBS to reach the desired amount of colony forming units (CFU)/mL.

### Glass cylinder methodology for topical application of bacteria

After resuspension, the bacterial strains were topically applied on the *stratum corneum* of the organotypic cultures using a glass cloning cylinder (Cat#070303-04, Bioptechs, Pennsylvania, USA) with an outer diameter of 6 mm (inner diameter of 4 mm). Cylinders were first washed with soap followed by disinfection with 70% and 100% ethanol (air-dried in flow cabinet). The cylinder was placed on top of the HEE, with the raw surface facing downwards in the middle of the insert, using forceps, leaving approximately 1 mm space at the edge of the culture area. 25 µL of bacterial suspension (or PBS only) was carefully pipetted inside the cylinder. During 4–5 h, the cultures were placed on a heated plate (37 °C) in the flow cabinet (without the lid) to allow the surface to become dry again. Afterwards, the cylinder was carefully removed and additional supplementation of culture medium (approximately 100 µL) in the basolateral compartment was required before returning cultures to the incubator at 37 °C and 5% CO_2_. A macroscopic view of the glass cylinder on top of the HEE is shown in Fig. 1B, whereas a schematic overview of the HEE culture schedule with bacterial exposure is depicted in Fig. 1F. During the culture experiments, samples of the culture medium were brought onto blood agar plates and incubated o/n at 37 °C to check for sterility.

Depending on the experimental design, the bacteria were applied at different time points of the ALI (day 7, 8, and 11) and HEEs were harvested after 6 h up to 13 days of culture. For the N/TERT-2G culture experiment, *S. aureus* ATCC 29213 was colonized at day 9 of the ALI.

To mimic the in vivo skin environment and to optimize culture conditions, HEEs inoculated with the SA-DUS-011 strain were also cultured at 32 °C (at the start of colonization, up to 10 days) at low relative humidity (dry atmosphere). This was accomplished by removing the water tray from the incubator. Of note, the culture medium in the basolateral chamber thereby evaporated faster requiring additional culture medium supplementation of 200 µL every day. Alternatively, the medium level could be increased with 500 µL to account for the evaporation and prevent the HEEs from running dry o/n.

The glass cylinder methodology was compared to a small droplet application (5 µL volume of bacterial suspension (SA-DUS-011 strain)) without the cylinder. The droplet was pipetted in the middle of the HEE (to minimize the risk of basolateral infections) and thereafter subjected to the same protocol as described above (37 °C and 32 °C).

### Topical application of antibiotics

Fusidic acid (FA, F0881, Sigma-Aldrich) was used as a narrow spectrum antibiotic known to combat *S. aureus* infections. Both *S. aureus* ATCC 29213 and the SA-DUS-011 strain were analyzed after the addition of FA in a concentration series. Immediately after the colonization of *S. aureus* (~ 4 h later, complete evaporation of PBS), 25 µL of FA (1% DMSO in water) was applied inside the same cylinder as used for the application of *S. aureus*. Again, the liquid was allowed to evaporate inside the flow cabinet (without lid on a heated plate, 37 °C) and the cylinders were carefully removed afterwards. The HEEs with *S. aureus* ATCC 29213 were subjected to 1, 10, and 100 µg/mL FA, incubated at 37 °C and 5% CO_2_ and harvested after 24 h (technical triplicates).

For a prolonged HEE culture experiment with the SA-DUS-011 strain, FA (10 and 100 µg/mL) was applied every other day using the sterile glass cloning cylinder on top of the HEE. Cultures were incubated at 32 °C (dry incubator) with 5% CO_2_ and harvested after 24 h (technical triplicates) and 8 days (technical quadruplicates) of colonization.

## Analysis method details

### Multi-parameter end point analysis of organotypic cultures exposed to bacteria

The polycarbonate filter supporting the organotypic culture was gently pressed out of the transwell by placing it up-side-down and using an 8-mm biopsy punch (BP-80F, KAI Medical). A 6-mm biopsy punch was used to sample the area that had been covered by the glass cylinder. The bacterial colonization area was macroscopically visible to the naked eye, which allowed the precise excision using the biopsy punch. Of this 6 mm sample, a 3-mm biopsy was punched and fixed for 4 h in 4% formalin for histological processing. The remainder of the sample was divided in two, with one part placed in 350 µL lysis buffer for total RNA isolation and the remainder in 250 µL PBS for CFU count, or in 500 µL PBS for microbial genomic DNA isolation for 16S rRNA gene sequencing. In summary, also depicted in the schematic image in Supplemental Figure S2B, samples were obtained for (i) tissue morphology and/or protein expression, (ii) bacterial growth, and (iii) host gene expression from one single HEE to minimize batch effects and increase assay throughput.

### Immunohistochemistry and confocal microscopy

Six micrometer paraffin sections were stained with hematoxylin and eosin (Sigma-Aldrich) or mounted with DAPI (4′,6-diamidino-2-phenylindole) fluoromount-G (Thermo Fisher Scientific) after deparaffinization. For immunohistochemical analysis, sections were first blocked with 5% normal goat, rabbit or horse serum in PBS for 15 min and incubated with the primary antibody for 1 h at room temperature or o/n at 4 °C (Supplemental Table S3). Thereafter, the sections were washed in PBS and subsequently incubated with biotinylated secondary antibodies for 30 min. Next, sections were washed again in PBS and incubated with avidin-biotin complex (1:50 avidin, 1:50 biotin in 1% BSA/PBS (v/v)) (Vector laboratories) for 30 min. Protein expression was visualized by color change due to the peroxidase activity of 3-amino-9-ethylcarbazole (AEC). The tissue was counterstained with hematoxylin, after which the sections were mounted with glycerol gelatin (Sigma-Aldrich, Cat No. 1002946952). For confocal microscopy, the primary antibodies goat anti-hBD2 and rabbit anti-SKALP were used in 1% BSA/PBS. As secondary antibodies, a donkey anti-goat Alexa Fluor 647 was used for hBD2 and a donkey anti-rabbit Alexa Fluor 594 was used for SKALP/elafin. All secondary antibodies (Molecular Probes, Eugene, OR) were diluted 1:200 in 1% BSA/PBS. Six micrometer paraffin sections were mounted in fluoromount-G (Thermo Fisher Scientific, USA) with DAPI (4′,6-diamidino-2-phenylindole). Image acquisition of immunofluorescence-stained tissue sections was performed by a ZEISS Axio Imager equipped with a ZEISS Axiocam 105 Color Digital Camera (Zeiss, Oberkochen, Germany). The ZEISS Axiocam 105 color is a compact five-megapixel camera (2560 × 1920 pixels) for high-resolution images with a 1/2.5” sensor. For confocal microscopy, the Zeiss LSM900 confocal laser scanning microscope objective 63 × numerical aperture 1.4, focal plane 1 mm, was used. Images were chosen as representative of the whole culture or biopsies and stored in CZI format.

### Keratinocyte RNA isolation and RT-qPCR analysis

RNA from the epidermal cells was isolated with the E.Z.N.A. Total RNA Kit I (OMEGA bio-tek) according to the manufacturer’s protocol. Isolated RNA was treated with DNaseI (Invitrogen) and used for cDNA synthesis using SuperScript IV VILO Master Mix (Invitrogen) and UltraScript 2.0 (PCR Biosystems) according to the manufacturer’s protocols. Subsequent real-time quantitative PCR (RT-qPCR) was performed using SYBR Green (Bio-Rad). qPCR primers were obtained from Biolegio (Nijmegen, The Netherlands) and depicted in Supplemental Table S4. Target gene expression levels were normalized using the house keeping gene human acidic ribosomal phosphoprotein P0 (*RPLP0*). The ΔΔCt method was used to calculate relative mRNA expression levels [53].

### Bacterial analysis

To isolate the bacteria from the organotypic cultures, the sample was homogenized/disintegrated in 250 µL PBS using a plastic micro pestle (Bel-Art, USA) in a 1.5-mL Eppendorf tube with convex bottom, by turning it around 10 times. Then, the suspension was completely homogenized using a needle (BD Microlance, 0.8 mm × 50 mm) and syringe (Henke-Ject, Tuberculin, 1 mL) by passing it 10 times. The homogenate was used to prepare a 10× dilution series and plated out on Columbia agar with 5% sheep blood. Plates were incubated at 37 °C either o/n at aerobic conditions or for 2 days at anaerobic conditions. CFUs were counted and corrected for dilution and harvesting method, considering that only a part (3/8) of the culture was used for counting.

### Dye penetration assay

To determine the time point of *stratum corneum* formation allowing bacterial colonization, 25 µL of 1 mM lucifer yellow (LY, Sigma-Aldrich) was applied inside a glass cylinder on top of the HEEs at various time points of the ALI culture (day 5 till day 8) and incubated for 2.5 h at 37 °C. After routine formalin fixation and embedding in paraffin, 6 µm sections were counterstained and mounted using DAPI Fluoromount-G (Thermo Fisher Scientific). LY was visualized at excitation wavelength of 488 nm using the ZEISS Axiocam 305 mono and a × 10 or × 40 objective.

### Statistical analysis

Statistical analysis was performed using GraphPad Prism 9.0 (https://www.graphpad.com). Each HEE culture experiment includes technical replicates from a single keratinocyte donor, unless specified otherwise in the figure legend.

For the RT-qPCR gene expression analysis, the raw ΔCt values were used. An unpaired t-test was performed to determine statistical significance between two groups. Paired (biological replicates) and unpaired one-way analysis of variance (ANOVA) was used for comparison between multiple groups followed by Tukey’s multiple comparison post hoc test.

To determine statistical significance for the CFU count data, unpaired nonparametric one-sided Mann‒Whitney *U* test was used.

## Results

### The prerequisites for bacterial colonization of organotypic skin in vitro

For bacterial colonization of organotypic skin and the study of host-microbe interactions, prevention of cell culture infection is crucial. Like in native intact skin, the *stratum corneum* of organotypic skin models should form a barrier preventing bacteria from penetrating the epidermis. Therefore, the start of bacterial inoculation heavily depends on the correct *stratum corneum* formation of the organotypic HEEs to discriminate bacterial colonization from invasive infection. The first appearance of lipid-rich *stratum corneum* layers that marks the time point of inoculation can be easily visualized by a tracer molecule, lucifer yellow (LY). For all primary keratinocyte donors (*N* = 8), LY was retained in the *stratum corneum* at day 7 of the air-liquid interface (ALI) culture, which was therefore considered as the starting point for bacterial colonization of HEEs in further experiments (Supplemental Figure S1A). To address the suitability of the HEE model for long-term bacterial exposed culture studies, the lifespan of the HEEs was monitored. Expression patterns of the proliferation marker Ki-67, differentiation markers keratin 10 (K10) and filaggrin (FLG) and antimicrobial peptide (AMP) SKALP/elafin remained normal [54] for 25 days. The number of *stratum corneum* layers, however, increased due to lack of desquamation in vitro (Supplemental Figure S1B). After 30 days, a reduced number of epidermal layers and loss of the granular layer was seen (Supplemental Figure S1C). Therefore, the window of opportunity for studying host-microbe interactions or intervention strategies in the herein presented HEE model system was estimated being 18 days: from the start point of bacterial inoculation at day 7 of the ALI to maximally day 25.

### Glass cylinder methodology for standardized topical inoculation of HEEs

In our efforts to optimize the bacterial application method for inoculating HEEs (from small to larger bacterial suspension droplets or complete coverage of the HEE), we were challenged by the labor-intensiveness, lack in reproducibility of bacterial colonization, high inter-individual variation between researchers, detrimental effects on epidermal morphology and most importantly frequent immediate infections (< 24 h of bacterial exposed culture) of the basolateral culture medium via the edges of the HEE. We therefore considered the utility of a glass cloning cylinder for topical application of the bacteria. The inert material minimally interacts with the bacteria or epidermis and allows easy sterilization. To quickly monitor the containment of liquid inside the cylinder at macroscopic level, we visualized the distribution of trypan blue on the HEE without and with the glass cylinder (Fig. 1A, B, respectively). Microscopic analysis after LY application indicated an equal distribution over the *stratum corneum*, containment of liquid within the cylinder area and foremost clean edges of the HEE (Fig. 1C).

Next, we investigated the effects of the glass cylinder and proposed vehicle (PBS) on the viability and structural integrity of the HEE. Prolonged immersion of organotypic epidermis is less desirable considering the detrimental effects on skin barrier formation and function [55, 56]. Indeed, covering HEEs with PBS for 24 h changed the expression of markers for epidermal proliferation (Ki-67) and terminal differentiation (FLG) (Fig. 1D). To reduce the time of liquid coverage of the *stratum corneum*, the cultures were placed on a laboratory hot plate (set at 37 ºC) without the lid of the transwell culture plate in the laminar flow hood to accelerate PBS evaporation. Thereby, the glass cylinder could be removed within 4–5 h before returning the culture plates to the incubator. After careful morphological analysis (Fig. 1E), this culture setup as depicted in Fig. 1F was used as the basis for all further experiments.

### Inoculation of HEE with pathogens and skin commensals

**Fig. 2:**
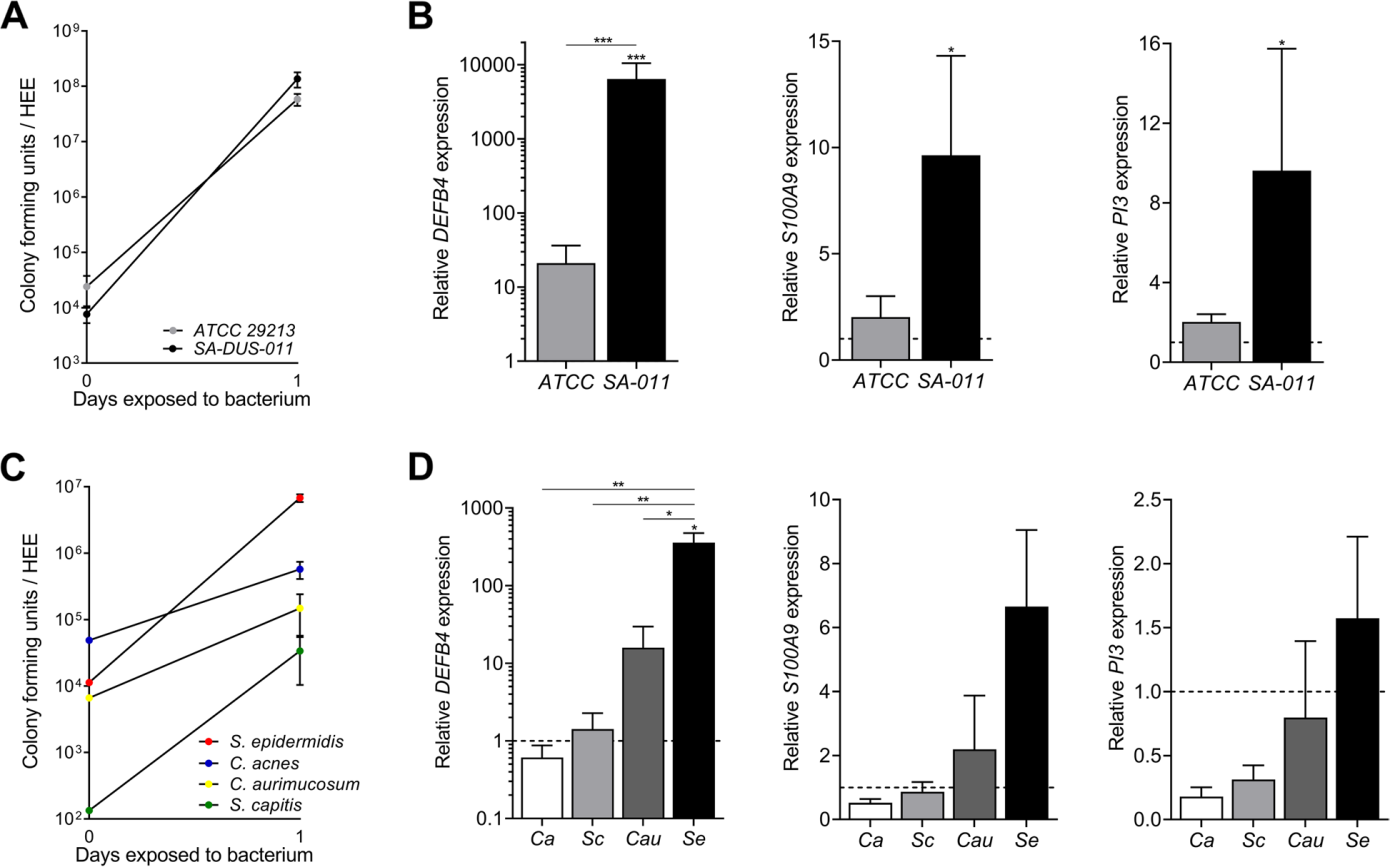
Colonization of HEEs with skin pathogens and commensals. **A** Growth and viability analysis by means of colony forming unit (CFU) count (input at 0 h) (biological *N =* 4) and **B** gene expression analysis of the antimicrobial peptides *DEFB4* (gene encoding hBD2), *S100A9* (MRP14) and *PI3* (SKALP/elafin) after 24 h of exposure to two *S. aureus* strains (ATCC 29213 and the clinical isolate SA-DUS-011) (biological *N =* 4, all controls set at 1). **C** CFU count (input at 0 h) (*N* *=* 3) and **D** gene analysis of *DEFB4, S100A9*, and *PI3* after 24 h of culture with skin related bacteria (*S. epidermidis = Se, C. acnes = Ca, C. aurimucosum = Cau, S. capitis = Sc*) (*N =* 3, control set at 1 (dashed line)). *p < 0.05, **p < 0.01. Mean ± SEM

For acquiring first proof-of-concept on our methodology, a bacterial suspension of the pathogen *S. aureus* (ATCC 29213, 10^4^ CFU in PBS) was added inside the glass cylinder, followed by a colonization period of 24 h. Whole epidermal tissue analysis (8 mm biopsy punch) showed a homogenous distribution of the bacteria on the *stratum corneum* in the middle part, whilst keeping the edges of the HEEs free from bacteria (Supplemental Figure S2A). Next, we used one single HEE for multi-parameter readout analysis (Supplemental Figure S2B). After 24 h of culture with two *S. aureus* strains (ATCC 29213 and a clinical isolate from an AD patient (SA-DUS-011)), CFU analysis indicated exponential bacterial growth reaching similar CFUs for both strains, with unaffected epidermal morphology (Fig. 2A, Supplemental Figure S2C). Remarkably, marker gene expression analysis of AMPs (*DEFB4, S100A9* and *PI3*), revealed a strong induction after culture with SA-DUS-011 (Fig. 2B). Also inflammatory mediators, here illustrated by *CCL20* and *IL1B*, were highly upregulated (Supplemental Figure S2D) in contrast to the laboratory ATCC strain.

To study the capability of aerobic, aerotolerant or facultative anaerobic skin commensals to colonize HEEs, *S. epidermidis*, *S. capitis*, *C. aurimucosum*, and *C. acnes* were applied and cultured for 24 h*.* CFU analysis indicated overall bacterial growth (Fig. 2C), albeit at different growth rates between the tested strains (Supplemental Figure S2E). No differences were observed in the morphological appearance of the HEEs exposed to different bacterial strains (Supplemental Figure S2F), yet expression levels of host defense marker genes were significantly different, and mostly highly induced by *S. epidermidis* (Fig. 2D, Supplemental Figure S2E and G). Importantly, no basolateral infections occurred during all HEE cultures as confirmed by plating culture medium onto blood agar plates.

### Prolonged HEE culture with S. aureus ATCC 29213

**Fig. 3:**
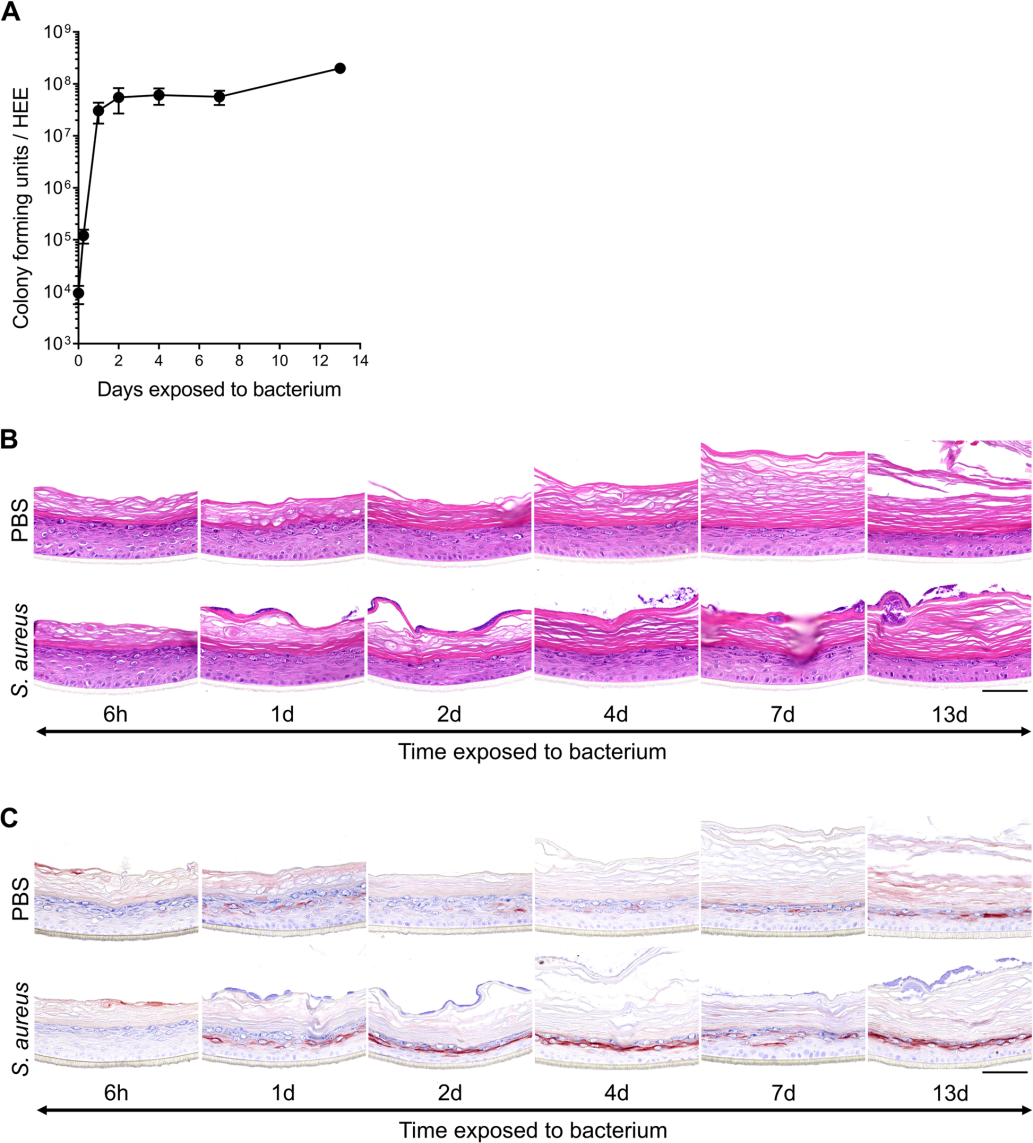
Prolonged HEE culture analysis after *S. aureus* ATCC 29213 colonization. **A** Colony forming unit (CFU) analysis of HEEs inoculated with *S. aureus* ATCC 29213 and harvested at different time points of culture up to 13 days (input at day 0). All data points represent *N =* 4 biological keratinocyte donor replicates, except for the 13 days culture (*N* *=* 1). **B** H&E and **C** SKALP/elafin staining of the HEE donor cultured for 13 days with *S. aureus* and its vehicle (PBS). Scale bar = 100 µm

Considering the favorable aerobic growth conditions for *Staphylococci* in HEE models and cell cultures in general, infections are expected upon long-term cultures if the glass cylinder does not effectively constrain the bacteria from leaking via the HEE edges, or when bacteria actively penetrate the *stratum corneum*. Being a commonly used human pathogenic strain, *S. aureus* ATCC 29213 was first selected for a prolonged 2-week culture period on top of the HEE. *S. aureus* quickly reached a maximum of approximately 10^8^ CFU within 24 h followed by a plateau phase during 13 days of culture (Fig. 3A). The growth and survival of *S. aureus* on the HEE was irrespective of the start inoculum, reaching maximum levels between 10^7^ and 10^8^ CFU after 20 h in all conditions (Supplemental Figure S3A). The epidermal morphology and protein marker expression for keratinocyte proliferation (Ki-67) and differentiation (FLG, involucrin (IVL)) of the HEEs cultured with *S. aureus* were comparable to control HEEs (Fig. 3B, Supplemental Figure S3B). Induction of SKALP/elafin protein expression was observed after 24 h of bacterial exposure and remained stable over time (Fig. 3C).

Accumulating *stratum corneum* layers due to lack of desquamation in vitro (Fig. 3B) could in principle hamper potential host-microbe interactions at later stages of the culture period. However, *stratum corneum* thickness did not influence bacterial growth and viability (Supplemental Figure S3C), nor did it hamper the induction of SKALP/elafin (Supplemental Figure S3D) when applying *S. aureus* at later stages of the ALI (day 11). Considering the popularity of the immortalized N/TERT keratinocytes in skin science as an alternative cell source for primary keratinocytes, we generated HEEs from the N/TERT-2G cell line which resulted in similar colonization rates as observed for primary keratinocytes (Supplemental Figure S4A-B). Again, in all experiments, no infections occurred during the short-term culture period.

### Epidermal infections after prolonged colonization by S. epidermidis and S. aureus

**Fig. 4:**
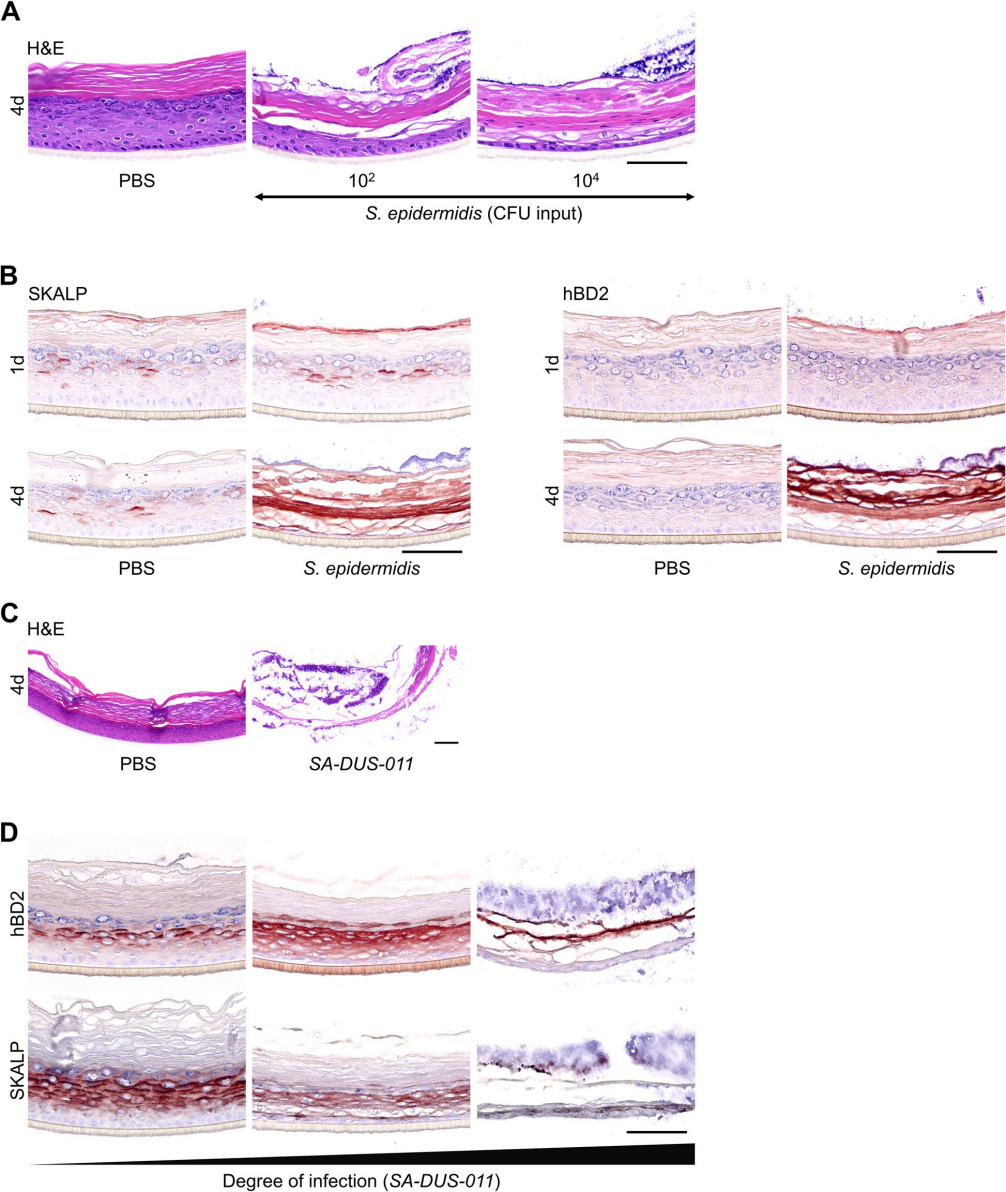
Epidermal infections caused by *S. epidermidis* and *S. aureus*. **A** *S. epidermidis* (10^2^ and 10^4^ CFU input) caused epidermal infections within 96 h of culture, visualized with H&E staining that revealed the structural damage and loss of granular layer compared to the control HEE (PBS). **B** Immunostainings of the AMPs SKALP/elafin and hBD2 showed induction of protein expression in case of an epidermal infection. **C** H&E staining of HEE colonized with the *S. aureus* clinical isolate SA-DUS-011 (10^4^ CFU input) for 96 h compared to the control HEE (PBS). **D** HEEs inoculated with SA-DUS-011, harvested at different time points of infection and stained for the AMPs SKALP/elafin and hBD2. All HEEs had multiple visible large yellow colonies on top of the *stratum corneum*. Only the culture medium of the first HEE was not infected yet, analyzed with a blood agar plate and o/n incubation at 37 °C. Scale bar = 100 µm

Commensal bacteria like *S. epidermidis* can become opportunistic pathogens causing skin infections [57] and may induce AD-like disease at high abundances [58]. Considering the strong host defense response we observed already after 24 h of HEE colonization (Fig. 2D, Supplemental Figure S2G), we evaluated the effects of a more prolonged culture with *S. epidermidis.* Epidermal infections occurred within 96 h, even at a minimal input inoculum of 10^2^ CFU. Structural damage of the epidermis, including loss of the granular layer, parakeratosis and reduced epidermal layers was observed (Fig. 4A). Strong induction of hBD2 and SKALP/elafin protein expression after 96 h (Fig. 4B) was subsequently accompanied by the confirmed presence of bacteria in the culture medium. Of note, no basolateral infections were observed after 24 h. However, the already high AMP levels at 24 h may have resulted from intracellular or invading bacteria in the epidermis not visible by light microscopy. However, confocal microscopy of immunofluorescent stainings did not reveal any presence of either *S. epidermidis* or *S. aureus* in the lower layers of the *stratum corneum* nor the living cells beneath in the epidermis (Supplemental Figure S5) at this time point, confirming a host defense response by bacteria-secreted factors through the *stratum corneum.*

Since the *S. aureus* clinical isolate (SA-DUS-011*)* also showed strong induction of host defense gene expression at 24 h, we also prolonged this culture, resulting in basolateral cell culture infections within 96 h (Fig. 4C). Prior to bacterial growth in the basolateral compartment, yellow colonies typical for *S. aureus* were macroscopically visible on the HEE surface after 48 h. Harvesting the SA-DUS-011 HEEs at different time points indicated various degrees of infection by upregulated AMP expression (hDB2 and SKALP/elafin) at the start of infection followed by structural damage to the epidermis (Fig. 4D). Similar results were obtained using N/TERT HEEs. Herein, epidermal infections were seen in 5/6 replicates after 72 h with concomitant upregulation of *DEFB4* (Supplemental Figure S4C). The induction of AMPs upon microbial exposure may thus be considered as an indicator for epidermal infections in vitro at later days, even when the epidermal morphology is still unaffected and basolateral culture medium and epidermis shows no signs of infection.

### Bacterial infections related to culture conditions

To address the influence of potential experimental artefacts (e.g., *stratum corneum* defects) from the cylinder application, the glass cylinder methodology was head-to-head compared with the application of a small volume of SA-DUS-011 in the middle of the HEEs [59–61]. In addition, to better mimic the natural growth conditions of bacteria on skin, physiologically relevant culture conditions (32 °C, dry atmosphere) were compared to the traditional cell culture conditions (37 °C, high humidity; warm and humid).

**Fig. 5:**
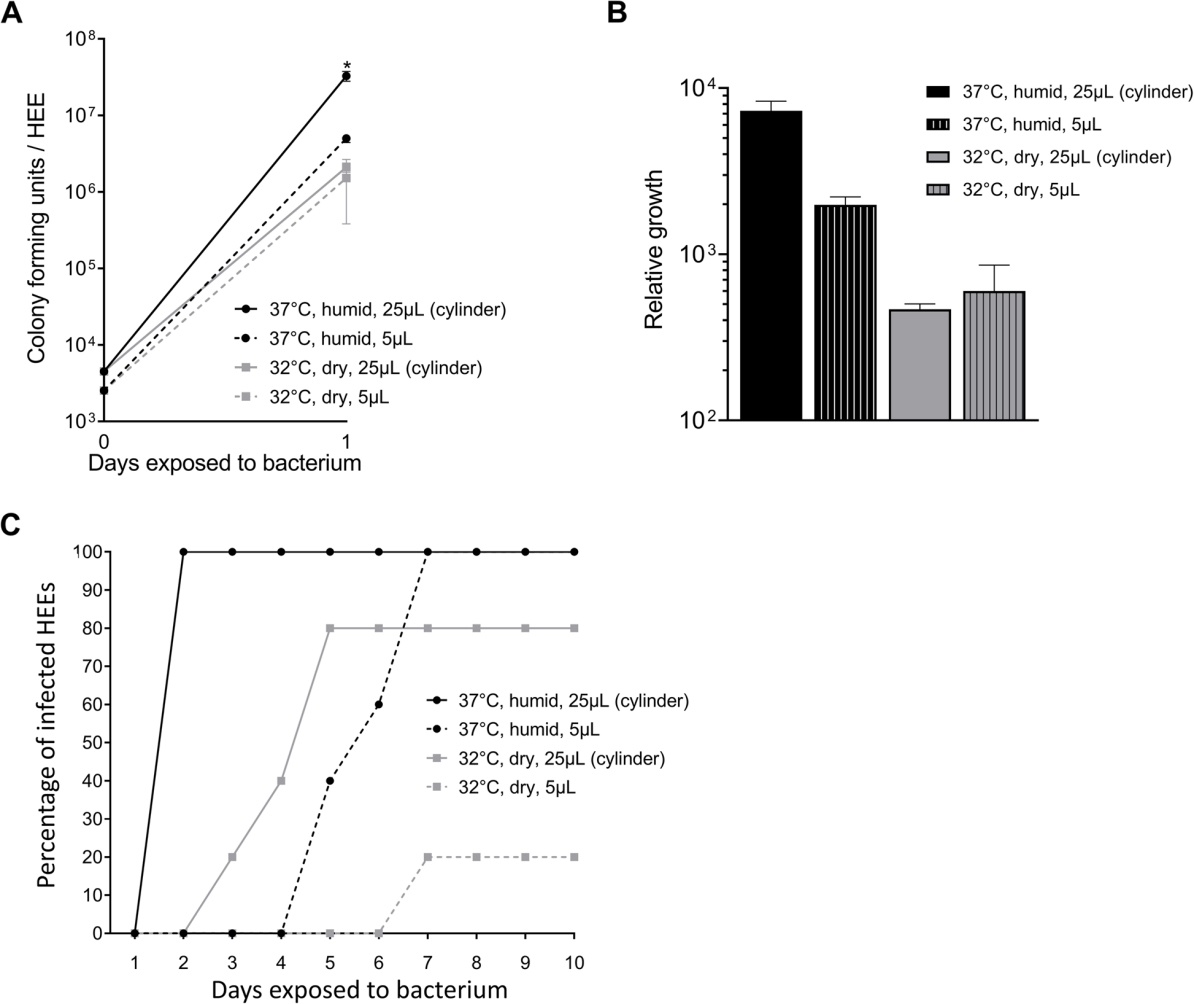
Bacterial infections using different culture conditions. **A** Colony forming unit (CFU) analysis and **B** relative growth of the *S. aureus* clinical isolate SA-DUS-011 after 24 h of colonization applying four different methods (glass cylinder methodology (25 µL) *versus* small droplet (5 µL) and 37 °C (humid) *versus* 32 °C (dry)) (*N* *=* 3 per method) (input at 0 h), *p < 0.05 (Mann-Whitney *U* test, CFU outcome of 37 °C glass cylinder method compared to the other methods). **C** Percentage of infected HEEs (*N* *=* 5 per method), cultured and exposed for up to 10 days with SA-DUS-011 applied using the four different methods

The large bacterial surface area in the cylinder in warm and humid conditions conferred significantly higher CFU count and relative growth than the droplet area and reached similar CFU counts as in previous experiments (10^7^–10^8^ CFU) (Fig. 5A, B). At 32 °C and dry conditions, a maximum CFU of 10^6^ per HEE was reached at both the droplet and cylinder application method, albeit the number of HEEs that became infected significantly differed between both application methods (Fig. 5C). Briefly, the smaller droplet area delayed infection onset in a warm and humid environment by at least 4 days, but could not prevent all HEEs becoming infected within 7 days after bacterial inoculation. At 32 °C and dry conditions delayed infection onset using the cylinder and even prevented infections in 80% of HEEs with a small (droplet) application area.

To further dissect the influence of temperature versus humidity on bacterial growth and infection rate, HEEs were also cultured at 32 °C in a humid environment. After 48 h, SA-DUS-011 caused epidermal infections in all HEEs that were incubated in humid conditions (of note: the infections started earlier at 37 °C compared to 32 °C). At 32°C and dry conditions, only 3 out of 8 HEEs became infected.

### Topical antibiotic inhibits the growth of S. aureus

Next to more fundamental studies on skin host-microbe interactions, organotypic 3D skin microbiome models could be of importance for research and development of pre-, pro-, and antibiotics to modulate the skin microbiome for therapeutic purposes. We implemented the cylinder methodology for the topical application of antibiotics using readout parameters for both host and microbe. Fusidic acid (FA) is used in clinical practice for the treatment of *Staphylococci* skin infections and herein chosen as a proof-of-principle intervention.

**Fig. 6:**
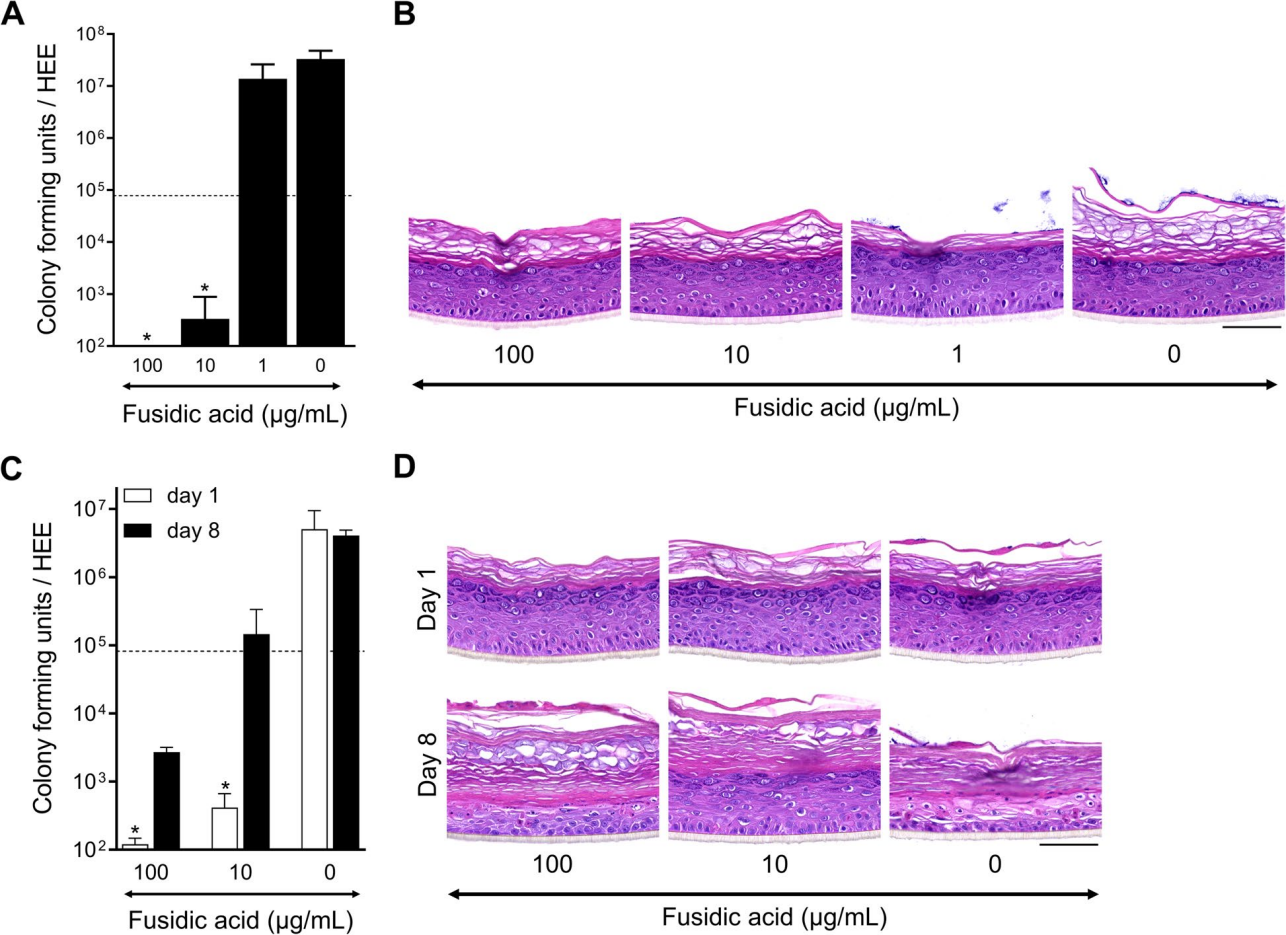
Fusidic acid inhibits the growth of *S. aureus* on HEEs. **A** Dose inhibiting effect via colony forming unit (CFU analysis) of HEEs topically applied with fusidic acid (FA, 1–10–100 µg/mL, 1% DMSO in water (0 µg/mL, vehicle)) 4 h after *S. aureus* ATCC 29213 colonization (dotted line: amount of CFUs at start of treatment) and harvested 24 h later (*N* *=* 3), and **B** H&E staining thereof. **C** CFU analysis on day 1 (*N* *=* 3 per treatment) and day 8 (0 µg/mL (*N =* 2) and 10–100 µg/mL (*N* = 4)), and **(D)** H&E staining of HEEs colonized with the *S. aureus* clinical isolate SA-DUS-011 subjected to the FA treatment protocol (10 and 100 µg/mL). HEEs were analyzed with a prolonged culture up to 8 days to study epidermal infections; 50% (2 out of 4) *S. aureus* HEEs infected after 96 h (FA applied at day 0, 2, 4, and 6) (of note, cultured at 32 °C (dry)). *p < 0.05 (Mann-Whitney *U* test, CFU outcome of fusidic acid dosages compared to the vehicle (0 µg/mL)). Mean ± SEM. Scale bar = 100 µm

Inhibition of *S. aureus* ATCC 29213 growth was observed in a dose dependent manner after a single dose of FA was added inside the cylinder directly after the initiation of *S. aureus* colonization, indicating the bacteriostatic effect of FA (Fig. 6A). In the morphological analysis, the lower amount of *S. aureus* colonies on top of the *stratum corneum* relate to the effective FA treatment. At the effective FA concentrations of 10 and 100 µg/mL, no morphological changes of the HEE were observed (Fig. 6B). Based on the aforementioned optimal culture conditions, FA efficacy was tested (10 and 100 µg/mL) on the *S. aureus* clinical isolate SA-DUS-011 using the glass cylinder and culturing at 32 °C and in a dry environment up to 8 days. At day 1, CFU analysis showed a strong reduction of *S. aureus* (Fig. 6C) indicative of the effective bacteriostatic effects of FA (bacteria were not completely killed, resulting in 10^5^ CFU on day 8 upon FA treatment every other day). During the following 7 days, 50% of the untreated *S. aureus*-colonized HEEs became infected after 4 days. The remainder of the untreated *S. aureus*-colonized HEEs that were harvested at day 8 showed severe epidermal damage (Fig. 6D) with high CFU counts (Fig. 6C) indicative of epidermal infections. FA treatment not only limited the bacterial growth, but also completely prevented infections and epidermal damage caused by *S. aureus* in HEEs.

## Discussion

We here present a technical advance for the topical bacterial inoculation of a 3D human epidermal equivalent (HEE) with a minimal risk of basolateral infections, whilst allowing in vitro studies on infectious virulent strains. This methodology using glass cylinders will be easily transferable to a wide variety of advanced organotypic skin [62, 63] or mucosal models [64], would be amenable for the application of diverse microbiota (bacteria [65, 66], viruses [67–69] and fungi [70, 71]) and can be used in every cell culture facility considering the various sizes and commercial availability, at low costs. We were able to increase the assay throughput by the large bacterial exposure area and thus obtaining multiple samples for various endpoint analysis from one single HEE. Furthermore, our model allows us to influence the cell culture environment to study infection *vs*. colonization. However, to more closely mimic the in vivo situation, more complex models involving immune cells and fibroblasts (full-thickness skin, ex vivo) and genetic predisposing factors need to be taken into account to fully comprehend biological mechanisms that underly host-microbe interactions in health and disease.

We generated both a colonization and infection model based on the single strain exposure of a fully developed epidermal model. While other bacterial exposed culture models to date induce an infection by making a wound [61, 63, 72, 73], we here showed that the *S. aureus* clinical isolate (SA-DUS-011) caused epidermal infections after colonizing an intact skin. Albeit similar growth rates and a high CFU output (10^7^–10^8^), the *S. aureus* strain ATCC 29213 did not infect the HEE within 2 weeks of culture nor did it induce the expression levels of any of the host defense markers. Based on these results, we consider the inoculum not being related to the AMP response, but rather depending on a strain specific effect and its secreted factors. Therefore, screening of various skin related bacterial species and using more than one strain per bacterium, ideally isolated from individual patients or volunteers, followed by whole genome sequencing [47], could relate virulence factors to the clinical features of the patient and host-microbe responses in vitro.

While here we present the model characteristics using single bacterial strains, the ultimate goal would be the application of whole skin microbiome samples or pre-designed microbial communities, as used in experimental animal models [13]. Yet, in vitro cell culture conditions have been shown to affect the stability of the commensal communities, skewing towards a dominance of aerobic bacteria after the culture period [47] and 16S or shotgun sequencing only includes information on relative abundancies whilst lacking information on bacterial viability. Methods to exclude bacterial DNA from dead cells, like propidium monoazide (PMA) [74], may provide a solution but require a labor-intense multi-step protocol and will be difficult to validate for the correct dosing of complex bacterial mixtures to avoid killing of microbes due to treatment.

The major advantage of a glass cylinder is the large colonization surface, allowing the collection of multiple samples, that we called “multiple parameter endpoint analysis”. A small droplet, as commonly used, prevents infection of the basolateral chamber, but will require multiple transwell inserts, large experimental setups, or cell culture formats (6–12well) [59, 62, 65, 66, 75–77]. Others completely cover the cell culture surface with bacterial suspension, but this requires immediate analysis or removal of non-adherent bacteria [71, 78–81]. Furthermore, when the set-up of experiments require multiple treatment steps of the equivalents, the cylinder provides a defined area wherein treatments can be applied after each other by equally distributed evaporation of the solutions, as we here showed for fusidic acid. This antibiotic prevented infections and maintained the epidermal morphology for at least 8 days of treatment, which is a novel finding compared to other antibiotic organotypic models [59, 78, 80, 82]. Although we found that the glass cylinder does accelerate the start of epidermal infections, a small droplet application also resulted in infections. Therefore, we value the utility of the glass cylinder and changed the culture environmental conditions (32 °C in a dry atmosphere) to delay the onset of infections and maximize the culture period and window of opportunity for interventions. By changing the cell culture environmental conditions and varying the application area of bacteria we leverage the opportunity to either study skin infection or colonization. Interestingly, we observed that under dry culture conditions, cultures located in the middle of the culture plate infected earlier than those in the outer rows, presumably due to higher humidity in the middle of the culture plate. Hence, only controlling the humidity in the cell culture incubator is not sufficient to fully standardize environmental conditions within the culture plate.

Modulation of microbiome composition and its effects might also be accomplished by changing host factors. We here showed that the use of the N/TERT-2G immortalized keratinocyte cell line is a suitable alternative for microbial colonization of HEEs since the epidermal structure is similar to that of primary keratinocytes [52]. In addition, it is the preferred cell type for genome editing and the use of a cell line instead of primary cells will reduce the biological variation. For example, knockdown of the differentiation protein filaggrin (*FLG*) showed increased colonization of *S. aureus* on top of the organotypic N/TERT model [79]. This correlation between FLG and microbial colonization is also observed in vivo for *S. aureus* [83, 84]. In addition, specific commensal species are underrepresented on *FLG*-deficient skin showing a reduction of gram-positive anaerobic cocci [37], that appear to harbor important AMP-inducing capabilities [41]. Furthermore, continued efforts in the optimization of culture conditions and protocols to better mimic the in vitro skin barrier to that of native skin [85, 86] will also affect the interaction between microbes and epidermal keratinocytes in organotypic model systems and as such, it will remain a challenge to compare results obtained between various models. Detailed information on the model characteristics (morphology, skin barrier function, cell sources, culture medium, microbial strain selection) are pre-requisites for studies that aim to investigate cell-host-microbe interactions in organotypic skin models.

In conclusion, our developed model system allows for easy utilization of organotypic human epidermal models for investigative skin microbiome research. This opens avenues into the application of more complex microbial cultures, the evaluation of specific pathogens in genotype-defined organotypic human skin models, and the screening of pre-, pro-, or antibiotic treatments therein.

### Supplementary Information

**Additional file 1: Supplemental Figure S1.** *Stratum corneum* formation and lifespan of HEEs. (A) H&E and DAPI staining of two HEE donors that were topically applied with LY for 2.5 h on different days of the air-liquid interface (ALI) to evaluate *stratum corneum* penetration (images represent eight biological keratinocyte donors). (B) Protein expression of the proliferation marker Ki-67, differentiation markers filaggrin (FLG) and keratin 10 (K10) and the AMP SKALP/elafin of a HEE at day 25 of the ALI. (C) H&E staining of HEEs harvested at day 25 and 30 of the ALI to investigate the lifespan of the culture. Scale bar = 100 µm.

**Additional file 2: Supplemental Figure S2.** Multi-parameter endpoint analysis, bacterial colonization, growth, and host defense response. (A) DAPI (white nuclei and colonies (white arrow)) and H&E (colonies indicated with black arrow) staining of HEE cultured for 24 hours with 10^4^ colony forming units (CFU) of *S. aureus* ATCC 29213 to visualize bacterial colonization and clean edges of the HEE. (B) Multi-parameter analysis for i) morphology and/or protein expression, ii) host gene expression and iii) bacterial growth. (C) H&E staining and (D) inflammatory gene expression (*CCL20* and *IL1B*) of HEEs colonized with *S. aureus* ATCC 29213 and the *S. aureus *clinical isolate SA-DUS-011 for 24 hours to analyze epidermal morphology (biological N*=*4, controls set at 1). (E) Logarithmic growth, (F) H&E staining and (G) inflammatory gene expression (*CCL20* and *IL1B*) after 24 hours of culture with skin related bacteria (*S. epidermidis = Se, C. acnes = Ca, C. aurimucosum = Cau, S. capitis = Sc)* (N*=*3, control set at 1).*p<0.05, ***p<0.001. Mean ± SEM. Scale bar = 100 µm.

**Additional file 3: Supplemental Figure S3.** Inoculum and *stratum corneum* thickness do not influence growth of *S. aureus* ATCC 29213. (A) Colony forming unit (CFU) count of HEEs inoculated with a concentration series (10^1^, 10^2^, 10^3^, 10^4^, 10^6^ and 10^7^ CFU) of *S. aureus* and harvested after 20, 28 and 44 hours of culture (N*=*2). (B) Normal epidermal protein expression after *S. aureus* colonization up to 13 days compared to the control HEE (PBS) shown with the proliferation marker Ki-67 and the differentiation markers filaggrin (FLG) and involucrin (IVL). (C) CFU analysis of *S. aureus* colonized at day 8 and day 11 (thick layer of *stratum corneum*) of the air-liquid interface (ALI) for 24 hours (biological N*=*5, input at day 0). (D) SKALP/elafin protein expression of HEE inoculated with *S. aureus* at day 11 of the ALI (thick layer of *stratum corneum*) in comparison with the control HEE (PBS) and cultured for 24 hours. Images represent N*=*5 biological keratinocyte donors. Scale bar = 100 µm.

**Additional file 4: Supplemental Figure S4.** HEEs generated with immortalized N/TERT cells and colonized with *S. aureus *strains. (A) Colony forming unit (CFU) analysis of N/TERT HEEs colonized with *S. aureus* ATCC 29213 and harvested after different time points of culture up to 7 days (each data point N*=*3), in comparison with primary human keratinocytes (grey line, biological N*=*4) and (B) H&E staining thereof. (C) Gene expression analysis of the antimicrobial peptide *DEFB4 *after 72 hours of culture with the *S. aureus *clinical isolate SA-DUS-011 (N=6). ****p<0.0001. Mean ± SEM. Scale bar = 100 µm.

**Additional file 5: Supplemental Figure S5.** Antimicrobial protein expression in HEEs colonized with *S. aureus *clinical isolate SA-DUS-011. Immunofluorescence detection of hBD2 (red signal) and SKALP/elafin (green signal) in HEEs using confocal microscopy. Nuclei of keratinocytes as well as bacteria are stained with DAPI (blue signal). Bacteria on top of the *stratum corneum* (upper panel *S. epidermidis*; lower panel *S.* *aureus*) are indicated with white arrows. Colocalization of hBD2 and SKALP/elafin in the upper layers of the epidermis is detected as a yellow signal in the merge column (only in HEEs colonized with *S.* *aureus*). HEEs were grown at 37°C and high humidity.

**Additional file 6: Supplemental Table S1.** Studies that used 3D organotypic skin models to investigate bacterial colonization, infection and host-microbe interactions [87–94].

**Additional file 7: Supplemental Table S2.** Antibodies used for immunohistochemistry.

**Additional file 8: Supplemental Table S3.** Bacterial strains.

**Additional file 9: Supplemental Table S4.** Primers for qPCR.

## Data Availability

Data or materials are available upon request by the senior author.

## Abbreviations

AD: Atopic dermatitis
ALI: Air-liquid interface
AMP: Antimicrobial peptide
BHI: Brain heart infusion
CFU: Colony forming units
DAPI: 4′,6-diamidino-2-phenylindole
FA: Fusidic acid
H&E: Hematoxylin and eosin
HEE: Human epidermal equivalent
IL: Interleukin
LY: Lucifer yellow
o/n: Overnight
PBS: Phosphate buffered saline
RT-qPCR: Real-time quantitative PCR
