# Supplementary material for: Novel methodologies for host-microbe interactions and microbiome-targeted therapeutics in 3D organotypic skin models
Source: Microbiome. 2023 Oct 17;11:227. doi: 10.1186/s40168-023-01668-x (PMC10580606; doi:10.1186/s40168-023-01668-x)
Supplement: Supplementary file 7 — Additional file 6: Supplemental Table S1. Studies that used 3D organotypic skin models to investigate bacterial colonization, infection and host-microbe interactions [87–94]. [file 40168_2023_1668_MOESM6_ESM.docx]

Supplemental Table S1: Studies that used 3D organotypic skin models to investigate bacterial colonization, infection and host-microbe interactions.

| Model | Cell source | Microbe(s) | Inoculation | Culture | Analysis | Reference |
| --- | --- | --- | --- | --- | --- | --- |
| HSE, colonization | NHEK (foreskin) | *S. epidermidis*  *C. acnes*  *M. furfur*  *S. aureus* | 10^2^ - 10^6^ | 72, 120 hours | CFUs, histology and TEWL | [[87](#_ENREF_87)] |
| HSE, colonization | NHEK (foreskin) | *S. epidermidis*  *S. aureus* | 10^4^ | 24 hours | RNA (microarray) | [[88](#_ENREF_88)] |
| HSE,  wound | NHEK | *P. aeruginosa*  *S. aureus* | 10^7^ | 24 - 72 hours | CFUs and histology | [[72](#_ENREF_72)] |
| HSE,  wound | NHEK (neonatal) | *P. aeruginosa*  *S. aureus* | 10^6^ | 3, 5, 7, 10, 24 hours | Histology | [[61](#_ENREF_61)] |
| HEE,  colonization | NHEK | *A. baumannii*  *A. junii* | 10^5^ | 72 hours | CFUs, histology and RNA (qPCR) | [[81](#_ENREF_81)] |
| HSE,  wound,  antibiotic | NHEK | *S. aureus* | 10^5^ | 24, 48 hours | CFUs, histology, IHC, RNA (qPCR) and ELISA | [[80](#_ENREF_80)] |
| HEE,  colonization | Skinethic | *S. aureus*  *S. epidermidis*  *C. acnes* | 10^7^ – 10^9^ | 24 hours | Biochemical assays, RNA (qPCR) | [[60](#_ENREF_60)] |
| HEE,  colonization | N/TERT | *S. aureus* | 10^5^ | 24 hours | CFUs, histology, IHC, RNA (qPCR) and ELISA | [[79](#_ENREF_79)] |
| HEE,  colonization | NHEK | *P. aeruginosa*  *S. aureus* | 10^6^ | 2, 24 hours | CFU, MTT | [[82](#_ENREF_82)] |
| *Ex vivo*,  colonization | Ovine biopsy | *D. nodosus* | 10^4^ | 28 hours (anaerobic) | Histology, FISH and ELISA | [[89](#_ENREF_89)] |
| HSE,  infection,  antibiotic | NHEK | *S. aureus* | 10^7^ | 24 hours | CFUs, Histology, LDH, RNA (qPCR) and ELISA | [[59](#_ENREF_59)] |
| HSE,  wound | NHEK | *S. aureus* | 10^4^ | 2 hours | SEM, CFU | [[90](#_ENREF_90)] |
| HSE,  infection | NHEK | *S. aureus* | 10^7^ | 24 hours | CFUs, histology, IF | [[62](#_ENREF_62)] |
| HSE,  wound | Labskin | *E. faecium*  *S. aureus*  *K. pneumoniae*  *A. baumannii*  *P. aeruginosa* | 10^5^ | 24, 48, 72, 96 hours | LESA mass spectra | [[73](#_ENREF_73)] |
| HSE,  Infection,  antibiotic | NHEK | *C. albicans* | 10^5^ | 24 hours | Histology, TEM, XTT assay, WB and immunoassay | [[71](#_ENREF_71)] |
| Ex vivo,  wound | Human biopsy | *M. sympodialis* | 10^6^ | 6 days | Histology, SEM, TUNEL, RNA, Immunoassay | [[63](#_ENREF_63)] |
| HSE, colonization | MatTek EpiDerm | *M. luteus*  *P. oleovorans* | 10^4^ – 10^6^ | 4, 8 days | CFUs, Gram stain, ELISA, RNA (qPCR, microarray), WB | [[75](#_ENREF_75)] |
| HEE,  colonization | Episkin | *C. acnes*  *M. restricta* | 10^5^ – 10^7^ | 72 hours | CFUs, histology, TEER, IF and SEM | [[91](#_ENREF_91)] |
| HEE,  colonization | NHEK (foreskin) | *C. acnes* | 10^5^ | 24, 48 hours | Histology, IF, LY, TEER, RNA and ELISA | [[66](#_ENREF_66)] |
| HSE,  colonization | EpiDerm | *S. aureus*  *P. aeruginosa*  *Microbiome* | 10^5^ | 18 hours | 16S and RNA sequencing, histology and IF | [[76](#_ENREF_76)] |
| HEE,  colonization, antibiotic | NHEK (foreskin) | *S. aureus*  *C. acnes*  *S. epidermidis* | 10^4^ | 1 hour | CFUs, histology, RNA (qPCR) | [[78](#_ENREF_78)] |
| HEE,  colonization | LabCyte Epi-Model (foreskin) | *S. aureus*  *S. epidermidis* | 10^3^ – 10^5^ | 48 hours | CFUs, LDH, ELISA, IF | [[65](#_ENREF_65)] |
| HSE,  infection | NHEK (foreskin) | *S. aureus* | 10^7^ | 2, 24, 48 hours | CFUs, histology, TUNEL | [[77](#_ENREF_77)] |
| HEE,  colonization,  therapeutics | Episkin | Microbial biofilm | 10^7^ | 24 hours | Histology, RNA (qPCR), Proteomics | [[92](#_ENREF_92)] |
| HSE,  colonization,  therapeutics | Phenion (foreskin) | *C. albicans* | 10^2^ | 24, 48 hours | CFUs, LY, histology, TUNEL, IF, ELISA | [[93](#_ENREF_93)] |
| HEE,  colonization | N/TERT,  NHEK | *S. aureus* | 10^5^ | 24, 48 hours, 8 days | CFUs, ELISA, Luminex | [[94](#_ENREF_94)] |

*HEE = human epidermal equivalent, HSE = human skin equivalent, NHEK = normal human epidermal keratinocytes, CFU = colony forming units, IF = immunofluorescence, qPCR = quantitative polymerase chain reaction, TEER = Transepithelial electrical resistance, LDH = lactate dehydrogenase, ELISA = enzyme-linked immunosorbent assay, LY = lucifer yellow, WB = western blot, TEWL = transepidermal water loss, IHC = immunohistochemistry, FISH = fluorescence in situ hybridization, LESA = liquid extraction surface analysis, TEM = transmission electron microscopy, SEM = scanning electron microscope, TUNEL = terminal deoxynucleotidyl transferase biotin-dUTP nick end labelling*
