# Supplementary material for: Novel methodologies for host-microbe interactions and microbiome-targeted therapeutics in 3D organotypic skin models
Source: Microbiome. 2023 Oct 17;11:227. doi: 10.1186/s40168-023-01668-x (PMC10580606; doi:10.1186/s40168-023-01668-x)
Supplement: Supplementary file 8 — Additional file 7: Supplemental Table S2. Antibodies used for immunohistochemistry. [file 40168_2023_1668_MOESM7_ESM.docx]

**Supplemental Table S2.** Antibodies used for immunohistochemistry

| **Target** | **Antibody clone** | **Antigen retrieval** | **Dilution** |
| --- | --- | --- | --- |
| Filaggrin (FLG) | FLG01, Thermo Fisher | Yes | 1:100 |
| Ki-67 | SP-6, Abcam | Yes | 1:200 (o/n 4°C) |
| Involucrin (IVL) | Mon150, van Duijnhoven *et al*. | Yes | 1:20 |
| Keratin 10 (K10) | DE-K10, Abcam | Yes | 1:100 |
| SKALP/elafin | 92-1, Schalkwijk *et al*. | No | 1:500 |
| Human β-Defensin-2 (hBD2) | ab9871, Abcam | No | 1:100 |
