# Supplementary material for: Novel methodologies for host-microbe interactions and microbiome-targeted therapeutics in 3D organotypic skin models
Source: Microbiome. 2023 Oct 17;11:227. doi: 10.1186/s40168-023-01668-x (PMC10580606; doi:10.1186/s40168-023-01668-x)
Supplement: Supplementary file 9 — Additional file 8: Supplemental Table S3. Bacterial strains. [file 40168_2023_1668_MOESM8_ESM.docx]

**Supplemental Table S3.** Bacterial strains

| **Strain** | **Identification number** | **Gram** | **Growth conditions** |
| --- | --- | --- | --- |
| *Cutibacterium acnes* | ATCC-6919 | positive | anaerobe |
| *Staphylococcus epidermidis* | ATCC-12228 | positive | aerobe |
| *Staphylococcus capitis* | Clinical isolate | positive | aerobe |
| *Corynebacterium aurimucosum* | Clinical isolate | positive | aerobe |
| *Staphylococcus aureus* | ATCC-29213 | positive | aerobe |
| *Staphylococcus aureus* | Clinical isolate | positive | aerobe |
