# Supplementary material for: Novel methodologies for host-microbe interactions and microbiome-targeted therapeutics in 3D organotypic skin models
Source: Microbiome. 2023 Oct 17;11:227. doi: 10.1186/s40168-023-01668-x (PMC10580606; doi:10.1186/s40168-023-01668-x)
Supplement: Supplementary file 10 — Additional file 9: Supplemental Table S4. Primers for qPCR. [file 40168_2023_1668_MOESM9_ESM.docx]

**Supplemental Table S4.** Primers for qPCR

| **HUGO**  **gene symbol** | **Protein** | **Forward primer**  (5’ → 3’) | **Reverse primer**  (5’ → 3’) |
| --- | --- | --- | --- |
| *RPLP0* | ribosomal phospoprotein P0 | caccattgaaatcctgagtgatgt | tgaccagcccaaaggagaag |
| *DEFB4* | human defensin-2, hBD2 | gatgcctcttccaggtgttttt | ggatgacatatggctccactctt |
| *CCL20* | chemokine (C-C motif) ligand 20 | tggccaatgaaggctgtga | gatttgcgcacacagacaactt |
| *IL1B* | interleukin-1β | aatctgtacctgtcctgcgtgtt | tgggtaatttttgggatctacactct |
| *S100A9* | S100 calcium-binding protein A9 | tgtggctcctcggctttg | gcgttccagctgcgacat |
